# Changing young people’s food-related behaviour: a socio-ecological perspective

**DOI:** 10.1017/S136898001900123X

**Published:** 2019-08

**Authors:** Anniza de Villiers, Mieke Faber

**Affiliations:** 1 Division of Research Capacity Development South African Medical Research Council, PO Box 19070, 7505 Tygerberg, South Africa; 2 Non-Communicable Diseases Research Unit South African Medical Research Council, Tygerberg, South Africa

The nutritional status of young children, adolescents and young adults has always been of interest to public health nutritionists. In the middle of the previous century the interest mostly focused on undernutrition and micronutrient deficiencies in under-resourced, often war-stricken countries of the world, where the environment was lacking in both quantity and quality of food^(^ ^1^ ^,^ ^2^ ^)^. Since then, industrialization and globalization have changed the food environment beyond anything that nutrition scientists could have foreseen. With the uprising of the obesity epidemic in both children and adults, the interest has shifted to food environments characterized by excessive availability of highly processed foods often high in energy and low in nutrient density. The taste and cost of these foods make them accessible and acceptable to both under- and well-resourced populations^(^ ^3^ ^)^.

The complexity of this highly evolved food environment is captured by the description of Swinburn and co-workers who describe these environments as ‘the collective physical, economic, policy and sociocultural surroundings, opportunities and conditions that influence people’s food and beverage choices and nutritional status’^(^ ^4^ ^)^. Nutrition researchers are trying to make sense of what changes are necessary and possible in this multifaceted environment to ensure better health for young people through their lifespan and into the next generation.

A socio-ecological framework, recognizing intrapersonal, interpersonal, organizational setting, community, environment and political influences, has often been used to study the factors that influence young people’s food-related behaviour. In 2012, Moore and colleagues presented an argument in this journal that ‘policy makers and health promotion practitioners are encouraged to identify complementary or, ideally synergistic policy components at multiple levels, rather than adopting an exclusive focus on intervening at any of the levels of influence on dietary behaviour’^(^ ^5^ ^)^. More specific to young people, following a study in Ecuador, Verstraeten *et al*.^(^ ^6^ ^)^ proposed a multilevel interactive framework that conceptualizes eating behaviour in adolescents as a function of both individual and environmental influences. They further highlighted the importance of context- and culture-specific key factors that mediate food choices. A recent *Lancet* report on obesity proposes an ‘inside out’ version of the socio-ecological model that is more suitable for explaining, according to the authors, ‘epidemics sweeping across entire populations’^(^ ^7^ ^)^. According to this model, individuals populate all levels of the human systems and continuously interact with natural ecosystems. This allows for three parts of the individual–environment interaction to become apparent: (i) the personal agency individuals have in making choices from the environments available; (ii) the influences of the environment on individuals’ choices; and (iii) the influence individuals have on changing environments and systems around them. Findings by Vogel *et al*.^(^ ^8^ ^)^, that high-agency interventions targeting individual psychological resources (e.g. self-efficacy, perceptions of healthy food affordability) combined with environmental interventions may be more effective in changing dietary behaviours than either intervention alone, provide support for the usefulness of this model in relation to the food environment. The model furthermore seems to be relevant to explain the interaction of young people with the food environment in and around schools.

Five articles^(^ ^9^ ^–^ ^13^ ^)^ in this issue of *Public Health Nutrition* highlight a range of issues related to the food environment and educational settings, and it is worthwhile to consider how the results of the different studies could contribute to the ongoing discourse about the food environment in and around schools and how individuals act in and interact with this environment.

An adequate intake of fruits and vegetables is considered essential for good nutritional health while a low intake of fruits and vegetables is associated with various health-related consequences including non-communicable diseases^(^ ^14^ ^)^. The intake of fruits and vegetables is therefore often used as an outcome measure when studying the relationship between the food environment and diet^(^ ^15^ ^)^. Van den Bogerd *et al*.^(^ ^9^ ^)^ did not collect any information on actual availability and cost of fruits and vegetables within the university environment but chose to focus on the perception of students. Although a large proportion of students perceived that enough fruits and vegetables were available in their university environment, intake thereof was low. Students felt that more affordable fruits and vegetables in the university canteen or supermarket would increase their fruit and vegetable intake. A study conducted in a web-based supermarket in the Netherlands provides some evidence that lower prices will stimulate purchases of fruits and vegetables^(^ ^16^ ^)^, pointing to a possibility that cost of food may support personal agency in healthy food selection.

An often-discussed aspect of the school food environment is the implementation of school policies as a vehicle to modify the school food environment to facilitate healthier food choices^(^ ^17^ ^)^. The paper by Godin *et al.* ^(^ ^10^ ^)^ in this issue provides some evidence that mandatory policies may be more effective than voluntary polices to restrict the availability of sugar-sweetened beverages in public schools. Yet, in this large cohort study in two provinces of Canada, the school environment had little association with the number of days sugar-sweetened beverages were consumed by secondary-school students, although schools in the province with the mandatory school nutrition policy had significantly lower availability of sugar-sweetened beverages. These results probably point towards the need for further research on the role of personal agency in the selection of sugar-sweetened beverages. Investigating the often-used alternative to sugar-sweetened beverages, the survey by Schermbeck *et al*.^(^ ^12^ ^)^ in this issue shows a lack of policies to regulate the availability of artificial sweeteners in the school food environment in the USA, making such foodstuffs available despite concerns by health professionals.

Inequality in exposure to different environments is reflected in a study by Assis *et al*.^(^ ^11^ ^)^. The authors report that areas with the highest levels of social deprivation in a city in Brazil had a lower density of establishments that sell food than the less vulnerable areas. The majority of establishments in both these areas sold predominantly unhealthy foods. Individuals may play an important role in influencing the environments and systems around them. In the school environment, students’ involvement in school initiatives supporting healthier food choices is considered important, yet a study in the Netherlands showed that students were reluctant to get involved as they felt their input was not valued^(^ ^18^ ^)^. On the provider side of the school food environment, a lack of support for school food-service professionals for creating healthier food environments is reported by Rida *et al*.^(^ ^13^ ^)^ in this issue.

Children and young people spend a considerable proportion of their time in an educational setting (school/university) and it therefore seems reasonable that the food environment in and around the educational setting should facilitate healthy food choices. Interventions within an educational setting that promote healthy eating should however consider that adolescents may have low risk perception of unhealthy eating and that they have a need to make independent food choices, particularly in the school setting, as was reported by Hermans *et al*.^(^ ^18^ ^)^. Furthermore, adolescents’ food choices generally are driven by taste, availability and accessibility, self-efficacy, financial constraints, time and convenience. The school food environment is but one component of a much larger environment and factors at household level and broader societal environments are likely to have a greater impact on children’s food choices. It is therefore important that population-level interventions are implemented together with school-based interventions, as recommended by Godin *et al*.^(^ ^10^ ^)^ in this issue.

The articles presented in this issue emphasize the multiple dimensions of the food environment and provide some insight into how these may influence young peoples’ choices and vice versa, as well as the personal power of young people to execute healthy food choices. The articles furthermore point to the need for public health nutritionists to engage with all the complexities of the school food environment and ensure that standardized and validated measures are available to capture these dimensions.
